# Applications of contemporary artificial intelligence technology in forensic odontology as primary forensic identifier: A scoping review

**DOI:** 10.3389/frai.2022.1049584

**Published:** 2022-12-06

**Authors:** Norhasmira Mohammad, Rohana Ahmad, Arofi Kurniawan, Mohd Yusmiaidil Putera Mohd Yusof

**Affiliations:** ^1^Center for Oral and Maxillofacial Diagnostics and Medicine Studies, Faculty of Dentistry, Universiti Teknologi MARA, Sungai Buloh Campus, Sungai Buloh, Malaysia; ^2^Center for Restorative Dentistry Studies, Universiti Teknologi MARA, Sungai Buloh Campus, Sungai Buloh, Malaysia; ^3^Department of Forensic Odontology, Faculty of Dental Medicine, Universitas Airlangga, Surabaya, Indonesia; ^4^Institute of Pathology, Laboratory and Forensic Medicine (I-PPerForM), Universiti Teknologi MARA, Sungai Buloh Campus, Sungai Buloh, Malaysia

**Keywords:** forensic odontology, human identification, dental age estimation (DAE), machine learning (ML), artificial neural network (ANN), deep learning

## Abstract

**Background:**

Forensic odontology may require a visual or clinical method during identification. Sometimes it may require forensic experts to refer to the existing technique to identify individuals, for example, by using the atlas to estimate the dental age. However, the existing technology can be a complicated procedure for a large-scale incident requiring a more significant number of forensic identifications, particularly during mass disasters. This has driven many experts to perform automation in their current practice to improve efficiency.

**Objective:**

This article aims to evaluate current artificial intelligence applications and discuss their performance concerning the algorithm architecture used in forensic odontology.

**Methods:**

This study summarizes the findings of 28 research papers published between 2010 and June 2022 using the Arksey and O'Malley framework, updated by the Joanna Briggs Institute Framework for Scoping Reviews methodology, highlighting the research trend of artificial intelligence technology in forensic odontology. In addition, a literature search was conducted on Web of Science (WoS), Scopus, Google Scholar, and PubMed, and the results were evaluated based on their content and significance.

**Results:**

The potential application of artificial intelligence technology in forensic odontology can be categorized into four: (1) human bite marks, (2) sex determination, (3) age estimation, and (4) dental comparison. This powerful tool can solve humanity's problems by giving an adequate number of datasets, the appropriate implementation of algorithm architecture, and the proper assignment of hyperparameters that enable the model to perform the prediction at a very high level of performance.

**Conclusion:**

The reviewed articles demonstrate that machine learning techniques are reliable for studies involving continuous features such as morphometric parameters. However, machine learning models do not strictly require large training datasets to produce promising results. In contrast, deep learning enables the processing of unstructured data, such as medical images, which require large volumes of data. Occasionally, transfer learning was used to overcome the limitation of data. In the meantime, this method's capacity to automatically learn task-specific feature representations has made it a significant success in forensic odontology.

## Introduction

Primary identifiers are the most reliable method of confirming identification (Jeddy et al., 2017). Fingerprinting, forensic odontology, and DNA profiling are examples of these identifiers. These methods differ in complexity, but they all have the same level of certainty. Forensic odontology is the simplest and fastest of these methods (Jain et al., 2020). It is a subfield of dentistry that focuses primarily on identifying a person's identity by analyzing the distinctive anatomical structure of the oral cavity (Divakar, 2017; Johnson et al., 2018). The primary applications of this field of study are in medico-legal investigations during a mass disaster, identifying accidental remains through the examination of dental records, and determining an individual's identity based on human remains (Hachem et al., 2020). In this subfield of forensic science, human identification is possible through the deceased body, which usually includes teeth and jawbones. This field is also crucial for identifying human remains after disasters like tsunamis, earthquakes, landslides, bomb blasts, etc., when bodies are so severely damaged and broken up that they can't be identified (Krogman and Isçan, 1986; Hinchliffe, 2011).

The dead bodies are usually identified visually by a close family member or a familiar person who knew that person throughout their life. This is frequently accomplished by visually observing the features of the face, several body options, or personal belongings. However, this technique becomes unreliable if the body options are lost due to post-and perimortem changes, such as decomposition or incineration. In such cases, visual identification may be prone to error. For instance, in cases related to criminal or suspected criminal cases, forensic experts may be needed to conduct the identification process through specific methods to analyze, identify and classify the physical evidence. For instance, cases related to criminal or suspected criminal cases may involve lots of laboratory work. The accuracy of human expertise is unquestionable as they are well trained, which means they are less likely to make a mistake. However, when a significant number of forensic evaluations are needed, it may lengthen the investigation process, eventually causing a burden on the experts and leading to human error. In addition, human identification associated with digital or radiological images may be helpful when clinical dental records are unavailable. The possible images may be acquired from dental x-rays, such as panoramic dental images and digital photographs usually used for analyzing human bite marks. Furthermore, Maber et al. (2006) stated that the radiological observation of the tooth development of permanent teeth among children aged between 4 and 14.9 years gives the most accurate dental age estimation, except for the third molars.

Artificial intelligence (AI) is widely defined as a tool which encompasses any techniques that enable computers to mimic human behavior and excel over human decision-making to solve complex tasks independently or with minimal human intervention (Janiesch et al., 2021). Hence, it is always concerned with a range of central problems, including environmental systems (Krzywanski, 2022), intelligent transportation systems (ITS) (Phillips and Kenley, 2022) and the earth's systems (Sun et al., 2022), and refers to a variety of tools and methods such as artificial neural networks, genetic algorithms, fuzzy logic and expert systems. The emerging computer systems based on intelligent techniques that support complex activities enable the automation system, especially in the medical industry. However, intelligent systems that offer AI often rely on machine learning (ML), which this approach describes the capacity of systems to learn from problem-specific training data to automate the process of analytical model building and solve associated tasks. In contrast, deep learning (DL) is the ML concept based on artificial neural networks (ANN). DL models outperform shallow ML models and traditional data analysis approaches for many applications. A convolutional neural network (CNN) is a prime example of DL, which uses the image as an input to the architecture. This approach has been getting attention from forensic and AI practitioners and is widely used in forensic odontology, especially in identifying individuals and sex dimorphism through radiological examination. However, due to various types of ML architectures applied in the previous study, which varied according to several factors such as its applications, type and amount of dataset used, study setting, and various inclusion/exclusion that varied from another study set by the authors, the best AI technology that can be applied in forensic odontology remain unknown.

A recent comprehensive review on the application and performance of artificial intelligence technology in forensic odontology has been conducted by Khanagar et al. (2021), which involves articles published between January 2000 and June 2020. However, there has been a significant increase in the number of publications on the use of ML and DL methods in forensic odontology within the last 2 years as Google launched TensorFlow 2.0 in June 2019, which declared Keras as the official high-level API of TensorFlow for quick and easy model design and training. The new technology was user-friendly and a highly effective interface for solving machine learning issues which influenced scholars, ML and DL practitioners to iterate on their experiments faster. This seems to be one of the factors behind the increase in publications regarding the application of ML in forensic odontology. This scoping review was conducted to assess the current ML and DL architecture regardless of any computer vision or image processing techniques used in forensic odontology. Thus, the primary research question that guides this review is “What are the current AI technology and its application performance in the field of forensic odontology?”

## Methods

A scoping review was conducted using the Arksey and O'Malley (2005) and updated by Joanna Briggs Institute (JBI) Framework for Scoping Reviews to clarify key concepts and identify gaps in the published literature. Scoping reviews map the available data from various sources to provide a broad overview of an ambiguous subject, in contrast to systematic reviews, which concentrate solely on a single question and review objective. In addition, because of the variety of recent ML and DL techniques used in forensic odontology among scholars, the authors decided to conduct a scoping review to identify research gaps of new knowledge and clarify the new concept of the proposed methods, which may also be valuable precursors to systematic reviews and can be used to confirm the relevance of inclusion criteria and potential questions, as stated by Munn et al. (2018).

The framework developed by Arksey and O'Malley has five components: defining the research question, identifying relevant studies, selecting studies, charting the data, and collating, summarizing, and reporting the results. This framework led to the development of the JBI protocol, which allows for systematic review and reporting while also making the process transparent (Peters et al., 2015). Furthermore, this review follows the Preferred Reporting Items for Systematic Reviews and Meta-Analyses (PRISMA-ScR) guidelines.

### Search criteria

The review was structured around a PCC question, an acronym for population, concept, and context. The JBI recommends using this type of question for scoping reviews (Peters et al., 2020). Table 1 shows the search criteria based on the “PCC” mnemonic.

**Table 1:** Description of the PCC elements.

**Population**	**Patients' diagnostic images related to oral and maxillofacial regions (clinical images, radiographs, CBCT)**
**Concept**	**AI-based models for human identification, age estimation, and sex determination**.
**Context**	**Performance between AI technology and traditional approach**

P, Population; C, Concept; C, Context.

The data for this study was gathered by searching for articles reported in the literature in renowned search engines, primarily PubMed, Google Scholar, Scopus, and Web of Science, published between March 2000 and June 2022. Based on that period, the databases were searched for the terms “artificial intelligence” OR “machine learning” OR “deep learning” OR “deep neural network” OR “convolutional neural network” AND forensic odontology. Table 2 summarizes the search terms used.

**Table 2:** Summary of keyword terms.

	**Keyword terms**
AI technology-related terms	Artificial intelligence Machine learning Deep learning Deep neural networks Convolutional neural network
Terms associated with Forensic Odontology	Human bite mark Age estimation Sex determination Dental comparison

### Study identification and selection

**Figure 1:**
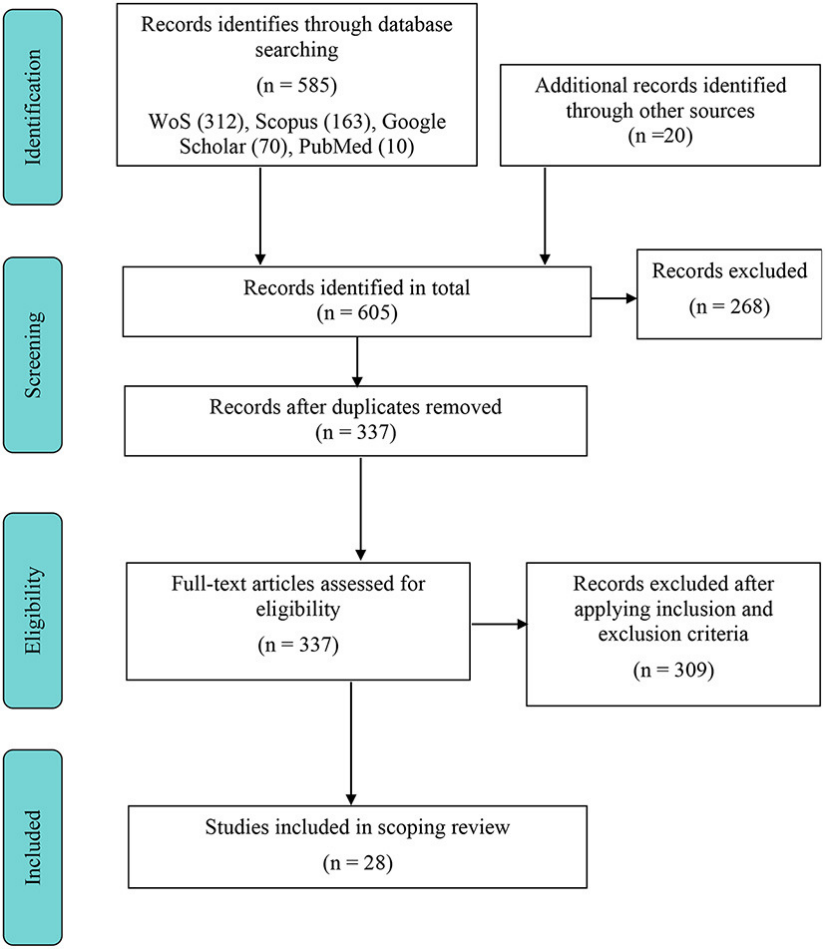
PRISMA-ScR flowchart of the study collection.

The relevance and importance of the selected study were evaluated based on its content and publication type. Therefore, only full-text research articles were included in this review. Following identifying articles in the abovementioned databases, they were imported into the EndNote X9 software (Thompson Reuters, Philadelphia, PA, USA), where duplicates were removed. Next, based on the titles and abstracts of the articles, the eligibility criteria were used to perform a preliminary screening. As shown in Figure 1 for the PRISMA-ScR selection process flow diagram, the full text of articles was then accessed to determine which articles were eligible for inclusion in the review. In contrast, editorial notes, reviews, and conference abstracts were excluded from this review.

### Eligibility criteria for the studies

#### Inclusion criteria

1. The article must concentrate on forensic odontology.
2. The AI technology employed in the study model should be explicitly stated.
3. There should be a clear statement of a predictive outcome.
4. The data sets utilized for training/validating or evaluating the AI model should be explicitly mentioned.

#### Exclusion criteria

1. Articles about subjects other than AI technology.
2. Articles that contain abstracts and no full-text articles.
3. Articles are written in languages other than English.

### Data extraction

A data extraction form was used to extract the available study details, such as the author(s), year of publication, and AI technology used. In addition, study characteristics such as study factor, image type, feature extractor/preprocessing method, algorithm architecture, evaluation, and findings were extracted. Finally, a narrative synthesis of the results was conducted to address the objectives.

## Results

Based on the initial search through selected databases using the keywords, we identified 605 articles, where 268 of them were excluded due to title, abstract, and duplicate removal screening. The remaining 337 articles were assessed for eligibility individually. Only 28 full-text articles fulfilled the inclusion criteria based on the final assessment. The comprehensive review process for the study collection is depicted in Figure 1. Table 3 summarizes the articles in the scoping review.

**Table 3:** Summary of all reviewed articles.

**Study factor**	**Author/year/ ML class**	**Image type**	**Feature extractor/Pre-processing method**	**Algorithm architecture**	**Evaluation**	**Findings**
**Human bite marks**	Mahasantipiya et al. (2012)**	Bite marks obtained from dental cast and captured by digital camera.	Manual measurements on the binary image	Multi-layer feed- forward NN	Mean Squared Error (MSE), Accuracy	The average accuracy is approximately 82%.
	Molina et al. (2022)****	Bite mark obtained from dental cast and scanned by the 3D scanner.	Manual measurements by human experts using Blueprint© software	N/A	ROC, AUC, ICC, Sensitivity, Specificity	Excellent inter-rater reliability (ICC >0.95), the highest area under the ROC curve (AUC) was obtained for the Euclidean distance of lower teeth rotation (AUC = 0.73)
**Sex determination**	Akkoç et al. (2017)*	Maxillary tooth plaster images	Gray Level Co-0ccurrence Matrix (GLCM)	RF Algorithm, SVM, ANN, Naive Bayesian, kNN	Classification Accuracy, Sensitivity, Specificity, ROC, AUC	RF algorithms outperform other ML algorithms with a 90% success rate.
	Akkoç et al. (2016)*	Maxillary tooth plaster images	Discrete Cosine Transform (DCT)	RF Algorithm	Classification Accuracy, Sensitivity, Specificity, AUC	The average classification was 85.166%, while the area under the ROC curve was 91.75%.
	Patil et al. (2018)**	Panoramic radiographs	Manual morphometric measurement by human expert using Digimizer Image analysis software	Feed-forward NN with backpropagation, Logistic regression	MSE, Mean Absolute Error (MAE), Of Determination (R^2^), R, LeastMean Square Error (LMSE), ROC, Sensitivity, Specificity	The overall accuracy of discriminant analysis was 69.1%, logistic regression was 69.9%, and ANN was 75%.
	Ortiz et al. (2020)**	Panoramic radiographs	Manual morphometric measurement by human expert	Logistic regression, ANN, Naive Bayesian, kNN	Discriminant Analysis, Training, and Testing Accuracy	Based on discriminant function, accuracy for females was 68.00% and 74% for males. Based on predictive analysis, the kNN model (0.937) and ANN (0.992) exhibit the best accuracy during the training phase, while during testing, NN (0.891) outperforms others.
	Esmaeilyfard et al. (2021) **	First Molar Teeth in Cone Beam Computed Tomography Images	Manual morphometric measurement by human expert	RF Algorithm, SVM, Naive Bayesian	Accuracy, Sensitivity, Precision, Specificity, ROC, AUC	Naive Bayesian was the best tool for sex classification, with an accuracy of 92.31%.
	Liang et al. (2021)***	Panoramic radiographs	mask-RCNN	ResNet34, Inception- ResNet	Mean Average Precision (mAP)	The proposed method surpasses all existing approaches, obtaining up to 59.62% mAP and 50.57% rank-1 accuracy.
	Milošević et al. (2021)***	Panoramic radiographs	DenseNet201, InceptionResNetV2, ResNet50, VGG16, VGG19, and Xception	A customized model which consists of a single 1x1 convolutional layer after feature extraction followed by the fully connected layer.	Model Accuracy (There are two models built: a family of models specialized for certain tooth types and a general model that can assess the sex from any tooth type)	The general model achieves an overall accuracy of 72.68%, while the specialized models achieve an overall accuracy of 72.4%.
	Nithya and Sornam (2022)***	Panoramic radiographs	NA	Five Convolutional layers, including a fully connected layer in the final layer.	Training Accuracy	The proposed CNN model exhibits better training accuracy (95%) than the VGG16 pre-trained model.
	Franco et al. (2022) ***	Panoramic radiographs	ROI was extracted by human experts using the Darwin V7 software package.	DenseNet121 associated with learning approaches: From scratch and transfer learning.	Model Accuracy, Classification Accuracy, ROC, AUC,	Transfer learning (82%) outperformed the from scratch architecture (71%). Also, females and males aged≥15 years were correctly classified at 87% and 84%, respectively, while females and males aged <15 were 80% and 83%, respectively.
**Age Estimation**	De Tobel et al. (De Tobel et al., 2017)***	Panoramic radiographs	Linear and Quadratic Discriminant analysis, Decision Trees, SVM, k-NN, Ensemble Classifiers	AlexNet	Rank-N RR, Mean Absolute Difference (MAD), Mean Linearly Weighted Kappa, ICC	The mean accuracy (Rank-1 RR) was 0.51, the mean absolute difference was 0.6 stages, the mean linearly weighted kappa was 0.82, and the mean ICC was 0.95. The novel method appears to be effective because the automated pilot approach used to stage the development of the lower third molar on panoramic radiographs resembled staging performed by human observers
	Merdietio Boedi et al. (2020)***	Panoramic radiographs	ROIs are cropped using Adobe Photoshop CC 2018 and segmented using built-in tools. Images are then grouped into three types: bounding boxes (BB), rough (RS), and full tooth segmentation (FS)	DenseNet201	Accuracy, MAD, Cohen's Kappa	FS dataset increased the staging allocation accuracy by 7% compared to BB. DenseNet201 was superior to AlexNet, as DenseNet201 improved the accuracy of stage allocation by 3%.
	Banar et al. (2020)***	Panoramic radiographs	Object detection: YOLO- Like CNN architecture Object segmentation: U-Net like CNN architecture	DenseNet201	Accuracy, MAE, Dice, Linearly Weighted Kappa	The current fully automated method for stage classification performed inferior to the semi-automatic approach proposed by Merdietio Boedi et al. (2020), with a stage classification accuracy of 54%, an MAE of 0.69 stages, and linearly weighted kappa of 0.79, respectively.
	Fan et al. (2020)***	Panoramic radiographs	ROIs which consist of five landmarks selected according to the forensic experience.	Customize CNN model: DENT-net	Recognition accuracy, false match rate (FMR), equal error rate (ERR), AUC	Rank-1 and Rank-5 accuracy of 85.16% and 97.74% were achieved, respectively. The AUC of the DENT- net was 0.996.
	Matsuda et al. (2020)***	Panoramic radiographs	NA	VGG16, ResNet50, Inception V3, InceptionResNet-V2, Xception, and MobileNet-V2	Accuracy	VGG16 model achieved the highest accuracy (100.0%) with pretraining and with fine-tuning.
	Lai et al. (2020)***	Panoramic radiographs	histogram equalization algorithm is adopted to adjust the brightness of the images.	Customize CNN model: LCANet	Recognition accuracy	Rank-1 and Rank-5 accuracy of 87.21 and 95.34% were achieved, respectively.
	Kim et al. (2021)***	Panoramic radiographs	ROIs consist of the maxilla and mandibular first molar of the right and left sides, manually extracted by the human observer.	ResNet152	Accuracy, AUC	The accuracy of the tooth-wise estimation was 89.05–90.27%. The AUC scores ranged between 0.94 and 0.98 for all age groups, indicating exceptional ability.
	Upalananda et al. (2021)***	Panoramic radiographs	Manual cropping was done by an expert on each stage's image of the mandibular third molar.	GoogLeNet	Accuracy, Sensitivity, Specificity	The overall accuracy of this method was 82.5%, and the accuracy at each stage of development ranged from 87.5% to 97.5%. The proposed study, which used GoogLeNet to look at different stages of development, is similar to a study done before on finding dental caries.
	Lee et al. (2020)***	Panoramic radiographs	Annotation of each tooth in the maxillae and mandibles was manually performed by expert.	mask R-CNN	F1-Score, Mean Intersection over Union (IoU)	The proposed method generated a mean IoU of 0.877 and an F1-score of 0.875 (precision: 0.858, recall: 0.893). In addition, the segmentation method's visual examination revealed that it closely matched the actual data.
	Kahaki et al. (2020)***	Panoramic radiographs	Projection-based transformation	Deep CNN with 5 convolutional layers and 2 fully connected layers	Model Accuracy	The results of the analysis show that the method is good at identifying images, which makes it possible for automated age estimation to be very accurate (81.83%).
	Mohammad et al. (2021)***	Panoramic radiographs	Dynamic Programming- Active Contour	AlexNet	Dice, Jaccard, ME, F-Score	The overall performance of the proposed classification approach to stage premolar development on panoramic radiographs was superior to the conventional method.
	Mohammad et al. (2022)***	Panoramic radiographs	Dynamic Programming- Active Contour	From Scratch	Accuracy, Training, Validation, and Testing Accuracy, Kappa Value	On the training, validation, and testing sets, the accuracy of the proposed model is 97.74, 96.63, and 78.13%, respectively. Although moderate agreement (Kappa value = 0.58) was achieved, no sign of the model's over-or under-fitting upon the learning process was seen.
	Milošević et al. (2021)***	Panoramic radiographs	DenseNet201, InceptionResNetV, ResNet50, VGG16, VGG19, and Xception	A customized model consists of a single 1x1 convolutional layer after feature extraction, followed by the fully connected layer.	R^2^, MAE, Model Accuracy	The fully automated DL model for complete panoramic radiographs has a mean absolute error of 3.96 years, a median absolute error of 2.95 years, and R^2^ of 0.8439.
**Dental comparison**	Mahdi et al. (2020)***	Panoramic radiographs	Manual annotation by expert dentist	Transfer learning with ResNet50 and ResNet101	F1-score, Accuracy, Precision, Recall	The average F1 score obtained is more than 0.97. So, the authors suggested that the proposed model could be a useful and reliable tool to help dentists do their jobs.
	Chen et al. (2019) ***	Digital dental periapical films	Manual annotation by expert dentist	Faster R-CNN with Inception Resnet version 2	Mean average precision (mAP), IoU, Precision, Recall	The results show that precision and recall are both greater than 90%, and the mean value of the IOU between detected boxes and ground truth is also greater than 91%.
	Miki et al. (2017) ***	Dental Cone-Beam CT images	Manual cropping is done by the experts	CNN with AlexNet	Classification accuracy, Detection rate	The accuracy of tooth detection was 77.4%, with an average false detection of 5.8 per image. According to the authors, the results show the potential utility of the proposed method for the automatic recording of dental information.
	Choi et al. (2022) ***	Panoramic radiographs	Manual annotation by the oral and maxillofacial radiologist using fully web-browser based labeling system developed by Digital Dental Hub (Seoul, Korea)	EfficientDet-D3, EfficientNet-B3	IoU, Precision, Recall	Natural teeth had an average precision of 99.1%, prostheses had an average precision of 80.6%, treated root canals had an average precision of 81.2%, and implants had an average precision of 96.8%.

* ML algorithm, **ANN, ***Deep Neural Network, ****Computational Technology.

###  General characteristics of the included studies for the scoping review

The dates of the publications ranged from 2010 to June 2022. Only one study was published in 2010. Another study was conducted after 6 years in 2016, three in 2017, one in 2019, nine in 2020, seven in 2021, and six in 2022. About 22 of the 28 articles were written in the last 2 years, which shows that interest in AI-based technology in forensic odontology applications started to rise in 2020.

**Figure 2:**
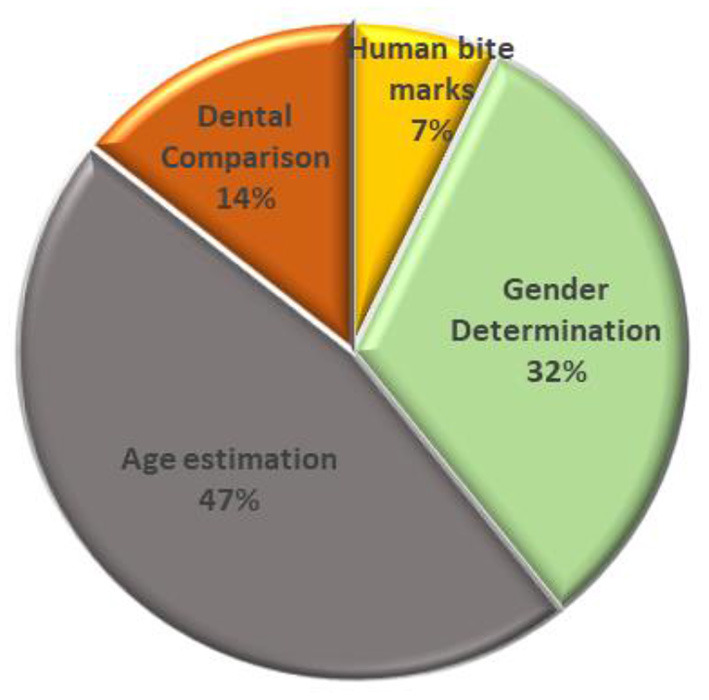
Forensic odontology classification.

As illustrated in Figure 2, forensic odontology can be classified into four significant thrusts: human bite marks, sex determination, age estimation, and dental comparison. With a total of 13 studies (47%), most studies focused on dental age estimation. In contrast, several publications involved dental practitioners (Johnson et al., 2018) and computer programming (Hinchliffe, 2011), and both approaches (Jain et al., 2020) in the image annotation stage or feature extraction. The second highest contribution was 38%, which is sex determination. Sex determination came in second (32%), with half of the publications employing a computer algorithm and half employing human experts to perform feature extraction, while one is not mentioned. Meanwhile, AI-based technology was rarely applied to human bite marks, where only two studies (7%) were included. Both studies employed human experts to annotate images during the feature extraction stage.

### AI-based method in forensic odontology

**Figure 3:**
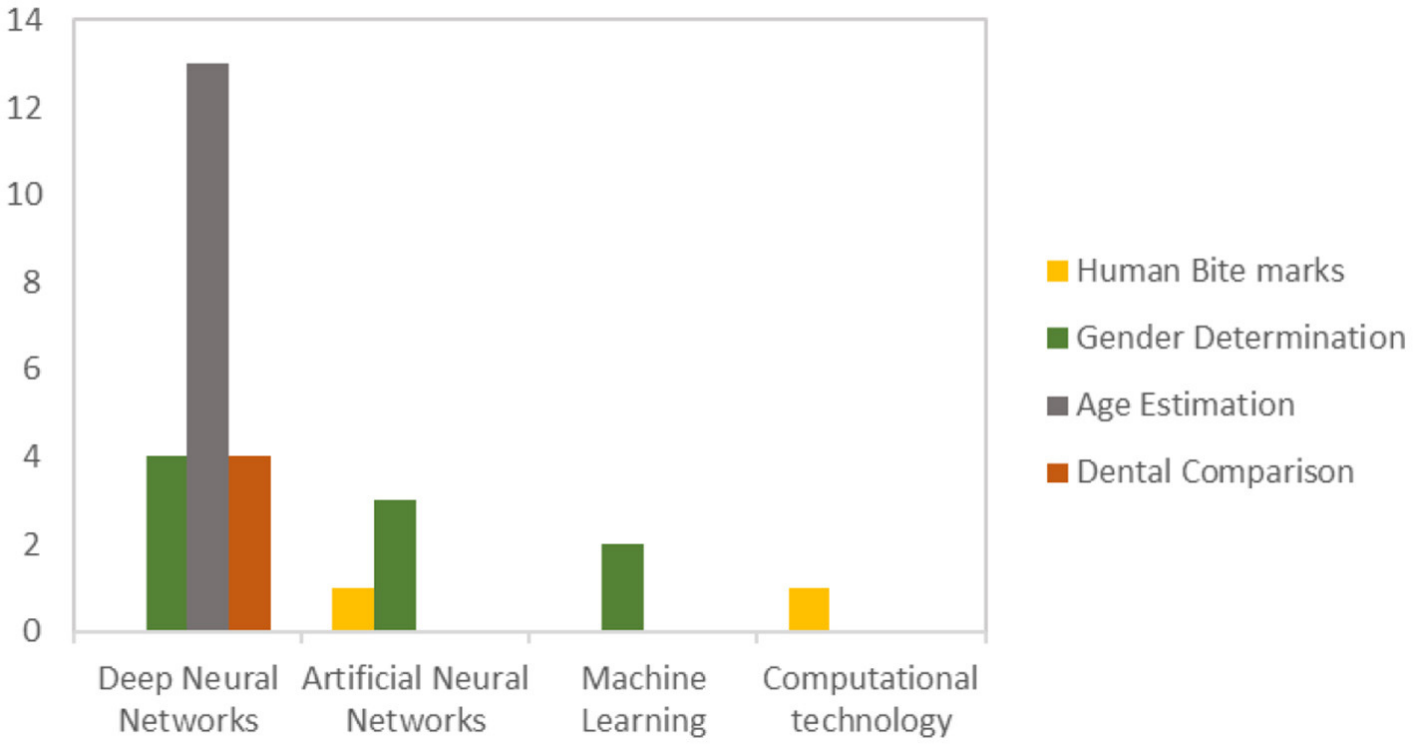
Publication of AI-based technology in forensic odontology.

Frequent AI-based technologies employed in forensic odontology include deep neural networks, artificial neural networks, machine learning, and computational technology. As illustrated in Figure 3, deep neural networks are the most frequently used in age estimation (Khanagar et al., 2021), sex determination (Johnson et al., 2018), and dental comparison (Johnson et al., 2018), with five of the studies recently published in 2022, following six in 2021, one in 2020 and 2019, and two in 2017. Next, ANN is the second most frequent method, mainly employed in sex determination (Divakar, 2017) and human bite mark analysis (Jeddy et al., 2017). In contrast, ML and other computational technologies were not employed in age estimation and were least used in sex determination (Jain et al., 2020) and human bite mark analysis (Jeddy et al., 2017).

## Discussion

### The conceptual distinction of the included studies for the scoping review

**Figure 4:**
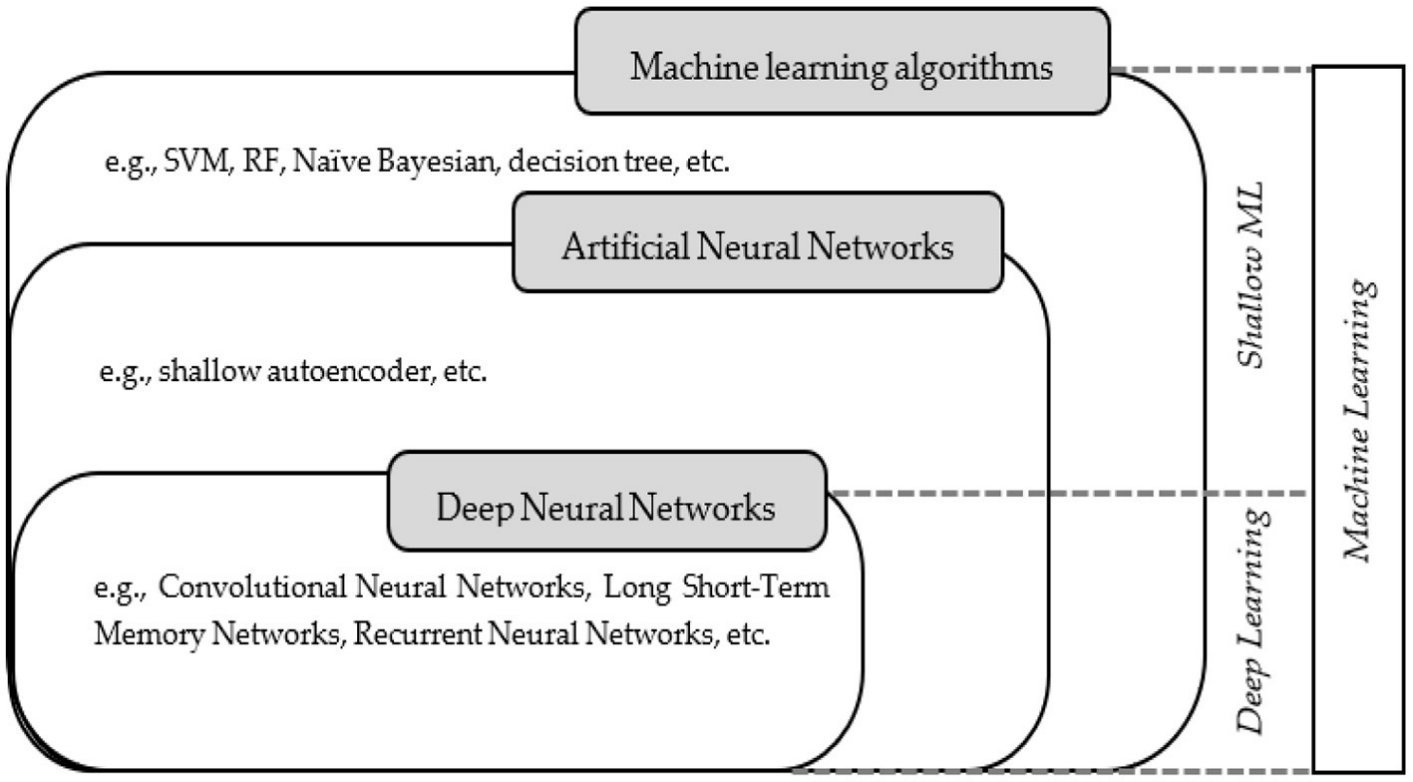
Venn diagram illustrating machine learning concepts and classes.

In this field, it is necessary to distinguish several relevant terms and concepts from each other. Three critical key terms that need to be well distinguished are machine learning algorithms, artificial neural networks, and deep neural networks. Despite the similarity between these terms, there are differences between them. The Venn diagram in Figure 4 depicts the hierarchical relationship between those terms.

Initially, AI research focused on hard-coded statements in formal languages, and a computer could automatically think about using rules for logical inference. Hence, according to Goodfellow et al., it is also known as the knowledge base approach (Goodfellow et al., 2016). However, Brynjolfsson and Mcafee (2017) stated that this approach has several limitations as it is difficult for humans to articulate all the implicit knowledge needed to carry out challenging tasks. Fortunately, ML is capable of overcoming these limitations. In terms of a class of tasks and performance measures, ML generally refers to the idea that a computer program performs better over time (Jordan and Mitchell, 2015). Hence, its main objective is to automate the development of analytic models to conduct cognitive tasks such as pattern recognition or object classification. Algorithms that can iteratively learn from training data specific to a given problem can be used to accomplish the goal. So, computers can find complex patterns and hidden information without being programmed (Aggarwal et al., 2022).

Machine learning shows good applicability when involving tasks related to high- dimensional data, such as classification, regression, and clustering. Moreover, it can improve reproducibility by learning from previous computations and extracting regularities from massive databases. Therefore, ML algorithms have been widely applied in many areas, such as image classification, speech and image recognition, or natural language processing. The ML algorithms are classified into three types (Saravanan and Sujatha, 2018): supervised, unsupervised, and reinforcement learning (RL). The overview of all ML types is presented in Table 4, and the comparison between these three types of ML is tabulated in Table 5.

**Table 4:** An overview of the type of machine learning.

**Type**	**Description**	**Example**
Supervised	ML is characterized by using labeled datasets to train classification or prediction algorithms (Singh et al., 2016). As the model receives input data, its weights are adjusted until the model is appropriately fitted. This step is included in the cross-validation process to ensure that the model does not over- or under-fit the data.	• Neural networks • Naive Bayesian • Linear regression, • Logistic regression • Random forest • Support vector machine
Unsupervised	It utilizes ML algorithms to analyze and cluster unlabeled datasets. Without human intervention, these algorithms uncover hidden patterns or data groupings (Shanthamallu and Spanias, 2021). Hence, training data only contains the variable x to find unique structural information, such as groups of elements with similar characteristics called clustering or data representations projected from a high-dimensional space into a lower one, known as dimensionality reduction (Bishop and Nasrabadi, 2006; Janiesch et al., 2021). Principal component analysis (PCA) and singular value decomposition (SVD) are two common techniques to reduce the number of features in a model. It is the best solution for recognizing images and patterns because it can find similarities and differences in data.	• Neural networks • k-means clustering • Probabilistic clustering techniques.
Reinforcement	Unlike the other two types, this ML model experiences the process of achieving the goal by itself, using the principle of trial and error to maximize reward. To achieve the goal, the system's current state must be described, a goal must be stated, and a list of permissible actions and information on the environmental constraints that will affect the results of those actions must be provided. This ML model has been successfully implemented in closed-world environments such as video games (Silver et al., 2018) and applies to multi-agent systems, such as electronic markets (Peters et al., 2013).	• Q-learning • State–action–reward– state–action (SARSA) • Monte Carlo • Deep Q Network (DQNN)

**Table 5:** Comparison of all types of machine learning.

**Comparison**	**Supervised**	**Unsupervised**	**RL**
Training data	Requires experts to label the data	Unlabeled data	Learn through interaction with the environment.
Preference	mapping of inputs and outputs	Clustering, identifying unique structural information, and mapping new patterns	AI (Behavioral learning)
Area	ML	ML	ML
Ideal approach	rely on the data and learning algorithm	rely on the data and its classification	Learn the ideal approach from experience
Illustration	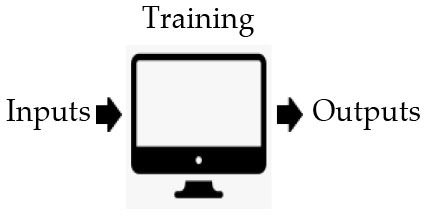	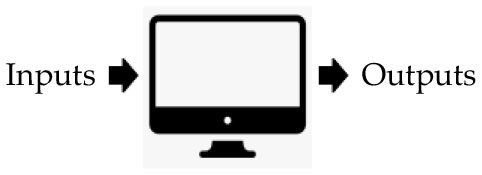	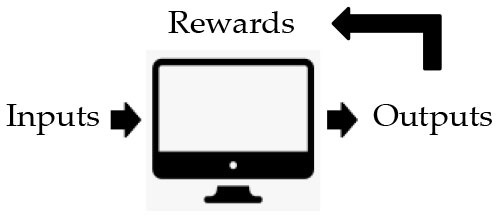

The field provides various classes of ML algorithms based on the learning task. Regression models, instance-based algorithms, decision trees, Bayesian methods, and ANNs are all different in their specifications and variants. The main distinction between the two approaches is that in supervised learning, the algorithm “learns” from the training dataset by making data predictions and adjusting for the correct answer iteratively. While supervised learning models are more accurate than unsupervised learning models, they require human intervention to label the data properly (Rudin, 2019). In contrast, unsupervised learning models work independently to discover the inherent structure of unlabeled data. It's essential to keep in mind that they still need human intervention to validate output variables.

Semi-supervised learning provides an advantageous balance between supervised and unsupervised learning (Reddy et al., 2018). During training, a smaller labeled data set is employed to guide classification and feature extraction from a larger unlabeled data set. Semi-supervised learning can solve the issue of insufficient labeled data for training a supervised learning algorithm.

Meanwhile, ANNs have gained wide attention due to their versatile structure, which enables them to be modified for a diverse range of situations among all three types of ML. Many functions, such as clustering, grouping, and regression, are made possible by ANN. For example, we can use ANN to group or sort unlabeled data based on similarities between the samples in the new dataset. In the case of classification, we can train the network on a labeled dataset to classify the objects in the dataset into several categories. The neural network's architecture and mechanisms are focused on the nature of the human brain. For example, humans use their brains to recognize patterns and distinguish various types of information, while NN can be trained to perform the same task on data. It consists of mathematical representations in which the biological neurons present in our brains inspire ANN. Each connection between neurons, like synapses in the brain, transmits signals whose strength can be amplified or attenuated by a weight constantly altered during the learning process (Janiesch et al., 2021).

The basic ANN architecture consists of input, hidden, and output layers. The input layer typically contains the input neurons that send the information/signal to the hidden layer. Then, in the hidden layer, the neurons will only process or fire the signals to the next neuron if those transmitted signals exceed specific threshold values determined by an activation function. This layer is useful for learning a non-linear mapping between input and output and can have zero or more hidden layers (Bishop and Nasrabadi, 2006; Goodfellow et al., 2016). Finally, the output layer generates final results such as image classification or binary input categorization. It should be noted that learning algorithms cannot learn the number of layers and neurons, the learning rate, or the activation function. Instead, they are the model's hyperparameters, which must be manually set or chosen by an optimization procedure.

Unlike simple ANNs, deep neural networks typically include sophisticated neurons of multiple hidden layers arranged in deeply nested network architectures. In this case, advanced operations such as convolutions may be used or have multiple activations in one neuron rather than a simple activation function. These features enable the deep neural network to process the raw input data and automatically learn the representation required for the corresponding learning task. This functionality, however, was not available in shallow ML, such as simple ANNs like shallow autoencoders and other ML algorithms like RF and decision trees. As some algorithms are innately interpretable by humans, shallow ML is identified as a white box. Most advanced ML algorithms, on the other hand, make decisions that can't be seen or understood. This makes them a “black box” (Janiesch et al., 2021).

According to LeCun (2015), deep neural networks outperformed shallow ML algorithms for most applications, including text, image, video, speech, and audio data. This is due to the ability of DL to deal with extensive and high-dimensional data. But in some situations, shallow ML can still do a better job (Zhang and Ling, 2018) than deep neural networks (Rudin, 2019) when there are few data points and low dimensionality. Hence, deciding which networks perform well is subjective and varies according to their applications. Nevertheless, various performance metrics can be used to evaluate the performance of ML algorithms. For example, metrics like log-loss, accuracy, confusion matrix, and AUC-ROC are some of the most common ways to measure how well classification works.

### Role of forensic odontology in human identification

Tooth eruption structures can be a valuable source of information for determining the victim's chronological age (Uzuner et al., 2017). The development of dentition is more closely related to chronological age. Human dentition has four distinct developmental periods (Uzuner et al., 2017); the emergence of deciduous teeth in the second year of life, the eruption of two permanent incisors and the first permanent molar between the ages of 6 and 8 years, and the emergence of other remaining permanent dentitions except for the third molars between 10 and 12 years, and the eruption of the third molars around 18 years of age. However, they may remain impacted (Holobinko, 2012). The radiographic evaluation based on dental development and mineralization is considered one of the most reliable methods for determining an individual's age among children and adolescents (Panchbhai, 2011). Through the radiological observation method, chronological age was calculated using the period between the date of birth and the day of the panoramic X-ray study.

Meanwhile, the size and shape of the victim's jawbone can be used to estimate the victim's sex. Sex determination using skull bone analysis is up to 90% accurate (Guyomarc and Bruzek, 2011). In mass disaster cases, when the victim's body is severely damaged to the point where visual identification is no longer possible, the remains of the individual's teeth, jawbones, and skull have proven to be the most valuable source for identifying the individual. The conventional way of estimating sex is through radiographic estimation, whereby the radiographs of the jawbones are considered more practical due to the non-destructive and straightforward method that can be applied to dead and living cases (Patil et al., 2018, 2020).

Examining the diseased person's soft tissues, which primarily include palatal rugoscopy and cheiloscopy (Nagare et al., 2018), is another method of identifying a person. Palatal rugoscopy studies the patterns on the palatal rugae to identify a person. Trobo Hermosa was the first to propose palatal rugoscopy in 1932. Because of its internal position, stability, and perennity, or the fact that it lasts throughout life, it is used in forensics for human identification (Ramakrishnan et al., 2015). On the other hand, the study of lip prints is called cheiloscopy. Lip prints can be identified as early as the sixth week of pregnancy. These prints remain unchanged. Lip prints are thus distinct patterns on the lips that aid in identifying a person (Tsuchihashi, 1974; Ramakrishnan et al., 2015; Nagare et al., 2018). One benefit of this method is that it costs less to examine, which makes it easier to check on both living and dead people (Indira et al., 2012).

Meanwhile, studies involving hard tissue indicate that dentine translucency seems to be one of the most reliable methods of determining an individual's chronological age. The progressive sclerosing of the tubules at the root causes the development of root dentine transparency. This process begins at the root apex and then moves coronally. A previous study reported that dentin translucency increases with age. Therefore, this method is reliable for individuals over 20 years of age when all their permanent teeth have erupted. Also, in forensic odontology, bite mark analysis is the best way to identify a person because injuries caused by teeth and left on things like skin have a unique pattern.

### AI-based method in forensic odontology

The application of the AI-based method in forensic odontology has proven to be a breakthrough in providing reliable information in decision-making in forensic sciences. Hence, we demonstrated in these papers that there are four primary areas which successfully employed AI technology at the moment: (Jeddy et al., 2017) human bite marks, (Jain et al., 2020) sex determination, (Divakar, 2017) age estimation, and (Johnson et al., 2018) dental comparison.

#### Human bite marks

Bite mark analysis is an important aspect and is the most prevalent type of dental evidence presented in criminal court. Matching bite marks to a suspect's dentition involves examining and measuring a person's teeth (Harvey, 1976). The principle of bite mark analysis is that “no two mouths are alike” (Gorea et al., 2014; Gopal and Anusha, 2018; Maji et al., 2018). The central doctrine of bite mark analysis is based on two assumptions: first, that human teeth are unique; second, sufficient detail of the uniqueness is rendered during the biting process to facilitate identification (Pretty and Turnbull, 2001; Lessig et al., 2006). Forensic odontologists can make appropriate decisions on personal identification and bite mark analysis due to the distinctiveness and uniqueness of human dentition. Bite marks can reveal individual tooth marks, a double-arched pattern, or multiple overlying bruises (Maji et al., 2018). In addition, bite marks can become deformed due to the skin's flexibility and elasticity. Bite marks can look different depending on how hard the bite was, where the body was, and how the upper and lower jaws were angled during the bite (Van der Velden et al., 2006; Osman et al., 2017).

Sörup (1926) published the first study on bite marks (Gill and Singh, 2015). Human bite marks are discovered when teeth are employed as weapons of rage, excitement, control, or murder (Pretty and Sweet, 2000). The imprints can also be found on the skin, stationery, musical instruments, cigarettes, and culinary items (Harvey, 1976). It can also be found in criminal cases, including homicides, quarrels, abductions, child abuse, and sexual assaults, as well as during sporting events, and is occasionally purposely caused to incriminate someone falsely (Van der Velden et al., 2006; Kashyap et al., 2015). Bite marks are a form of dental identification in and of themselves. It is now recognized that bite marks provide details comparable to fingerprints.

The usual term used in bite mark analysis is the victim, which indicates the recipient of the bite mark, and the perpetrator is the person who caused the bite marks (Chintala et al., 2018). Unlike bite marks on the body, which are usually caused on purpose, the offenders inadvertently leave bite marks on food at the crime scene. Hence, to identify the offender, dental casts of suspects are prepared and matched using dental material. The proper analysis of bite marks can prove the involvement of a specific person or persons in a meticulous crime (Kashyap et al., 2015). West et al. (1987) believed that bite marks on human skin could be experimentally created to a level that could be compared to bites delivered in aggressive or life-threatening situations. However, more research utilizing living subjects to explore a variety of experimental situations is required. Identifying, recovering, and analyzing bite marks from suspected biters is one of forensic dentistry's most unique, complex, and sometimes difficult challenges (Kashyap et al., 2015; Maji et al., 2018; Rizwal et al., 2021).

In contrast, to bite marks, analyzing the suspect's dentition includes measurement of individual teeth' size, shape, and position (Levine, 1972). Overlays are used in almost every comparison method (American Board of Forensic Odontology, 1986). Hand tracing from dental study casts (Sweet and Bowers, 1998), wax impressions (Luntz and Luntz, 1973), xerographic images (Dailey, 1991), the radio-opaque wax impression method (Naru and Dykes, 1996), and the computer-based method (Sweet et al., 1998; Kaur et al., 2013) are all methods for producing overlays from a suspect's dentition. Sweet and Bowers (1998) investigated the accuracy of these methods for producing bite mark overlays and concluded that computer-generated overlays produced the most accurate and reproducible exemplars. Intercanine distance (ICD) measurements are one part of the study since impressions of the front teeth are usually the most visible and most likely to be measured (Kashyap et al., 2015).

However, the use of ML methods in human bite mark analysis is still in the intermediate phase. Most previous methods, from manual to semi-automatic to fully automatic approaches, focus on computer vision systems that utilize image processing algorithms (Chen and Jain, 2005; Van der Velden et al., 2006; Flora et al., 2009). One of the reasons why this field is not getting a lot of attention from scholars and professionals is the growing doubts about how accurate bite marks can be used as evidence in court. Assumptions about the ability of bitemark comparisons to correctly identify the source of a disputed bite mark have progressed from widespread skepticism to pervasive credulity, with a growing return to skepticism (Saks et al., 2016). This growing skepticism stems from the realization that the field is built on a weak foundation of scientific proof, with a lack of valid evidence to support many of the assumptions and statements made by forensic odontologists during bite mark comparisons (Pretty and Sweet, 2001b; Bush and Bush, 2006; Franco et al., 2015) and that error rates by forensic dentists are possibly the highest of any forensic identification area of expertise still employed (Saks et al., 2016). Besides, the unsatisfactory nature of skin as a substrate for the registration of tooth impressions is one factor that raises doubts about the value and scientific validity of comparing and identifying bite marks (Council, 2009). The bite marks on the skin are easily changed over time and disrupted by skin elasticity, unevenness of the biting surface, swelling, and healing. Hence, these characteristics may strictly restrict the validity of forensic odontology (Janiesch et al., 2021).

However, Mahasantipiya et al. (2012) published a preliminary study on bite mark identification using ANN. The study aims to develop a ML model with high-performance accuracy and to overcome human bias during the analysis. The inclusion criteria include no missing lower and upper anterior teeth or fixed orthodontic appliances. Bite mark samples are then collected using the standard dental wax in five different biting positions. The bite marks of these samples were captured using the digital camera before the preprocessing algorithm. Selected features of the bite marks were chosen to undergo the learning process through the designed ML model. This study shows that the trained networks provided good matching accuracy. Although the accuracy of the proposed ANN was not so high, it shows that this approach has potential and should be investigated further to improve performance. Also, the authors suggested training the ML model on more features of the bite marks that could make it work better.

Although this pattern-matching evidence lacks the scientific foundation to justify continuing admission as trial evidence, most forensic odontologists believe that bite marks can demonstrate sufficient detail for positive identification (Saks et al., 2016). Molina et al. (2022) recently proposed a semi-automated analysis of human bite marks using two different software packages with the new intervention of computer software. DentalPrint generates biting edges from 3D dental cast images, while Biteprint is used to characterize the biting edges. The performance of the identification procedure was evaluated using the ROC curve. The authors reported that the highest area under the ROC curve (AUC) obtained for the Euclidean distance of lower tooth rotation was 0.73. Hence, their proposed method to measure lower tooth rotation may be helpful in identifying individuals responsible for bitemarks and may be relevant in forensic cases. In addition, this study established a new benchmark for future human bite mark analysis studies.

#### Sex determination

Sex determination is required when information about the deceased is unavailable. In the case of accidents, chemical and nuclear bomb explosions, natural disasters, crime investigations, and ethnic studies, determining a person's sex becomes the priority in the process of identification by a forensic investigator. Usually, forensic experts face a significant challenge when determining sex from skeletal remains, significantly when only body fragments are recovered. Using teeth and skull characteristics, forensic odontologists can help other experts determine the sex of the remains, as male and female teeth have different characteristics, such as morphology, crown size, and root length. In addition, the skull pattern and characteristics of the two sexes differed. Therefore, this will aid forensic odontologists in determining the sex of the remains.

There are several techniques for sex estimation, including odontological and anthropological methods. Both methods include various metric and non-metric variables and biochemical analyses (Capitaneanu et al., 2017). For example, dental methods for studying sexual dimorphism can be based on the morphology and measurements of teeth and other tissue structures, such as cheiloscopy (Karki, 2012; Kinra et al., 2014; Kaul et al., 2015), the palatal rugae (Bharath et al., 2011; Thabitha et al., 2015; Gadicherla et al., 2017), the mandible (Hu et al., 2006; Vinay et al., 2013), and sinuses (Kanthem et al., 2015; Akhlaghi et al., 2016). On the other hand, anthropological methods for figuring out a person's sex use the shape and size of bones like the skull, hip, sacrum, scapula, clavicle, sternum, humerus, and femur to confirm the individual's sex (Durić et al., 2005; Krishan et al., 2016).

Since automation trends in the medical field have been getting wide attention, computer science techniques such as ML, ANN, and DL are promising methods that can automate the conventional method and enhance reproducibility. Several studies have been published using ML techniques for sex determination. Akkoç et al. (2016) proposed a fully automated sex determination from individuals' maxillary tooth plaster model images. The image acquisition process is done before the segmentation and classification step. First, a standard image is obtained by fixing the camera angle on top of the mechanism and equipping it with cube-shaped light sources to absorb light from all directions. Based on the RGB color channel of the standard image, channel B provides significant features compared to others. Image segmentation includes converting color to a binary image, followed by morphological operations, which include binary dilation and erosion. Finally, the segmented plaster image was transformed into a gray-level image for feature extraction using a gray-level co-occurrence matrix (GLCM) method. Extracted features are then classified using the RF algorithm. Results show that the RF algorithm gives the highest classification accuracy compared to other methods such as SVM, ANN, and kNN. The authors made improvements by using the local discrete cosine transform (DCT) in extracting features and the RF algorithm for classifying images (Akkoç et al., 2017). Based on the 10-fold cross-validation, the average classification accuracy was 85.166%, and the area under the ROC curve was 91.75%

**Figure 5:**
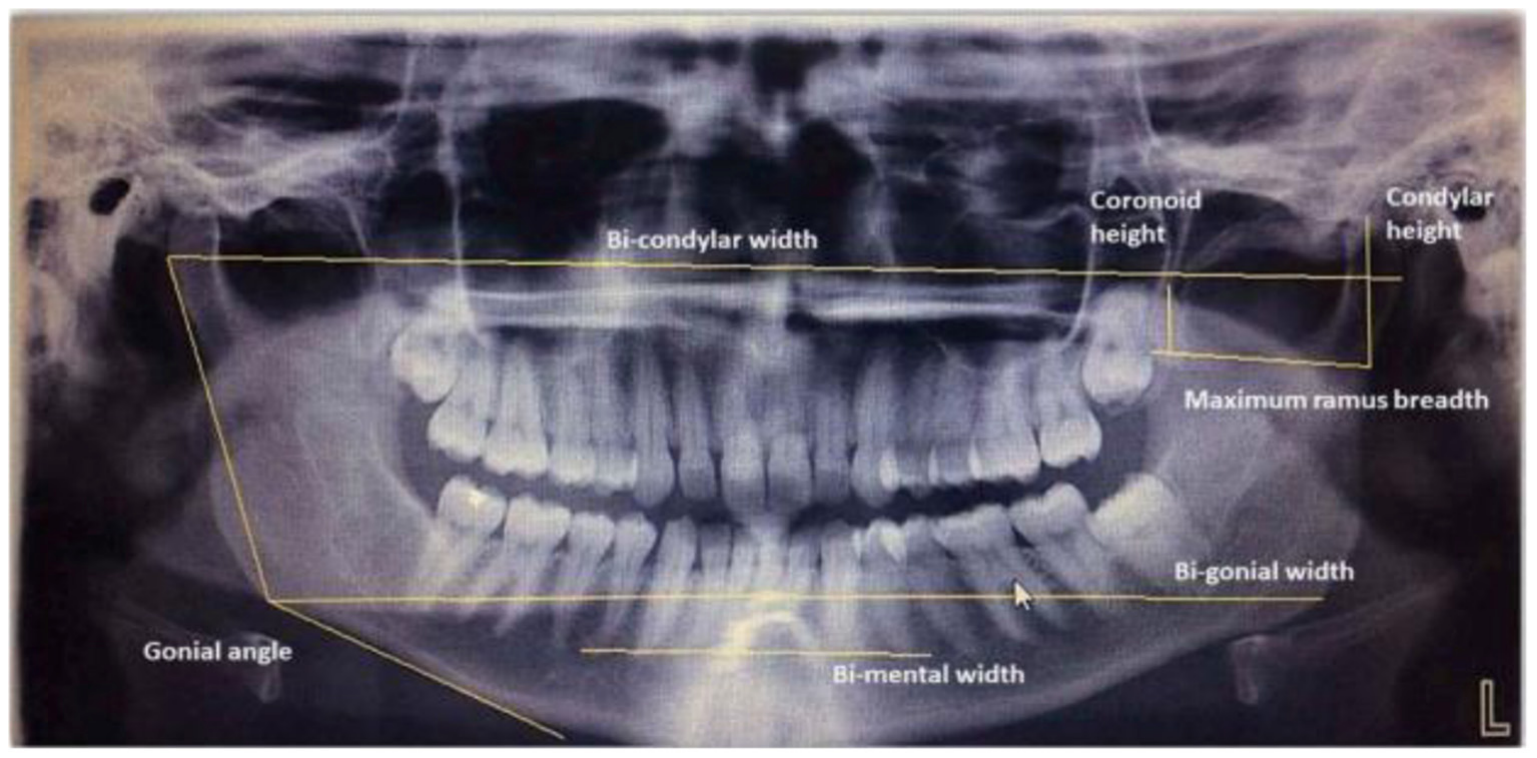
Morphometric parameter measurements done on digital panoramic radiographs by Raj and Ramesh (2013), Kumar et al. (2016), and More et al. (2017).

Meanwhile, a study using mandibular morphometric parameters that used digital panoramic radiographs has been proposed by Patil et al. (2020). Seven morphometric parameters were selected based on the previous studies (Raj and Ramesh, 2013; Kumar et al., 2016; More et al., 2017) for evaluation. Figure 5 shows the measurement of morphometric parameters done on digital panoramic radiographs. A feed-forward neural network with a backpropagation learning algorithm was proposed in this study. The NN model consists of an input layer, two hidden layers, and one output layer, where 70% of the dataset was assigned for training, 15% for validation, and 15% for testing. Based on the three analyses done on morphometric parameters, ANN analysis had a higher overall accuracy of 75% than Logistic Regression and Discriminant Analysis, both of which had an overall accuracy of 69.9%.

**Figure 6:**
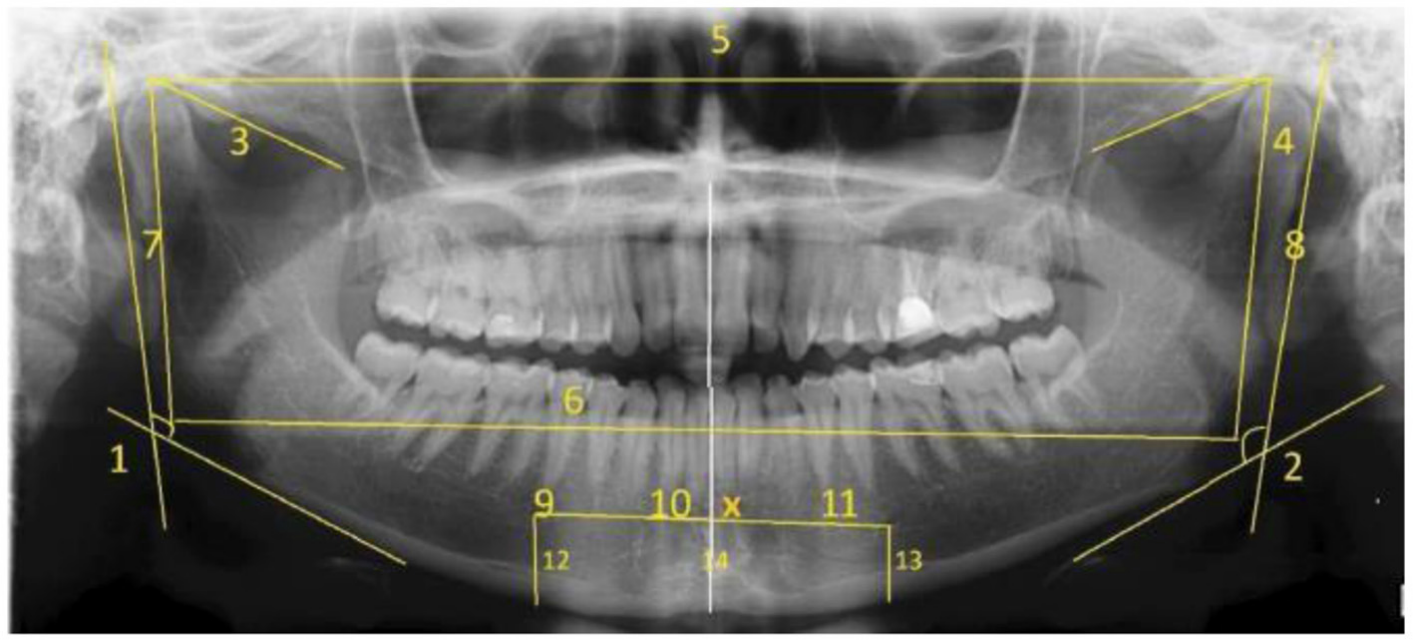
Measurement of morphometric parameters marked on panoramic radiographs (Ortiz et al., 2020). R, Right; L, Left; AMD, right mandibular angle; C, Condyle; Co, Coronary process; Go, Gonys; FM, Mental Foramen; PSM, Medium Saginal Plane; Me, Mento; BMD, Mandible Base; X Me, Me Intersection Point; PSM, Me.

Similar research has been published by Ortiz et al. (2020), but different morphometric measurements are done on panoramic dental radiographs. Figure 6 shows the measurement of parameters on panoramic radiographs where each number marked on the image is defined as follows:

- AMD (D)
- AMD (E)
- C-Co (D)
- C-Co (E)
- C-C
- Go-Go
- C-Go (D)
- C-Go (E)
- FM – FM: FM - PSM (D)
- FM – PSM (E)
- FM – FM x PSM
- FM – BMD (D)
- FM – BMD (E
- X Me

The authors performed the feature classification in this research using five different ML models: KNN, NN, stochastic gradient descent (SGD), Naïve Bayes, and logistic regression. Based on the predictive analysis, the KNN model exhibits the highest training accuracy of 93.7%, while NN shows the highest testing accuracy of 89.1%. Another promising ML model for sex classification was proposed by Esmaeilyfard et al. (2021) using the first molar teeth in Cone Beam Computed Tomography (CBCT) images. Feature extraction is performed before the classification. Nine parameters were measured in the centre of the corrected sagittal and coronal sections. These parameters are the roof, floor, and height of the pulp chamber, as well as marginal enamel thickness and dentin thickness at the height of contour (HOC), tooth width, and crown length in both buccolingual and mesiodistal aspects. This study experimented with three different classifiers: Naïve Bayesian, RF, and SVM. Based on the 10-fold cross-validation, Naïve Bayesian gives the best result, with an average accuracy of 92.31%.

Meanwhile, a recent publication employing DL methods for sex classification has been reported by Liang et al. (2021), where a CNN algorithm was proposed in which two pre-trained models are adopted: ResNet-34 (He et al., 2016) and Inception-ResNet (Szegedy et al., 2017). Based on the proposed method, the accuracy of Inception-ResNet is superior to the other pre-trained models, in which 59.62 and 50.57% were achieved in terms of mAP and rank-1 accuracy, respectively.

Another DL approach applied to the panoramic radiographs has been proposed by Milošević et al. (2021), in which several pre-trained models were tested: DenseNet201 (Huang et al., 2017), InceptionResNetV2 (Szegedy et al., 2017), ResNet50 (He et al., 2016), VGG16, VGG19 (Simonyan and Zisserman, 2015), and Xception (Chollet, 2017). Hyperparameter tuning was done to determine the best DL model for the study. VGG16 shows the most successful model based on tuned hyperparameters, as it can achieve a classification accuracy of up to 77%.

Early this year, in January 2022, Nithya and Sornam (2022) reported a detailed study with clear explanations of deep convolutional neural networks (DCNN) using dental x-ray images. The authors created their own CNN architecture, which comprises five layers of sequential networks, where the final layer is fully connected. In this paper, the hyperparameter values are listed. For example, batch size 50, Adam optimizer, and categorical cross-entropy loss are assigned to the algorithm. As a result, 95% accuracy was obtained. The authors stated that their proposed method was superior to the existing one, which utilized transfer learning through a pre-trained model of VGG16 (Ilić et al., 2019).

Although numerous tools for sexual dimorphism based on morphological dental traits in identifying sex are available, challenges still restrict their performance. Franco et al. (2022) presented a preliminary study on the applicability of an ML setup to distinguish males and females using dentomaxillofacial features from a panoramic radiograph dataset. Employing two different CNN architectures, one was the network built from scratch, and the other was transfer learning associated with DenseNet121, the authors reported that the classification accuracy of the transfer learning architecture was superior to the from-scratch model, which is 82% and 71%, respectively. The authors suggest that as this study aims to understand the discriminant power of dental morphology to distinguish between males and females, the current findings should not be applied in practice. However, the authors listed the hyperparameters, allowing other scholars to improve the ML architecture and prediction performance.

#### Age estimation

Extensive longitudinal studies in this field have influenced scholars and professionals to go beyond the conventional approach. A more sophisticated age estimation process can be done using computer science techniques with less human intervention. The automation process of age assessment proved to have exact reproducibility and be equally prominent, just like the conventional method. The most common method of dental age estimation based on tooth development is based on Demirjian et al.'s (1973) staging system, which uses digital panoramic dental imaging to estimate an individual's chronological age based on the mineralization of seven permanent left lower teeth. This method is also suitable for determining dental maturity states, whether the individual with a known age is advanced or delayed, rather than predicting an unknown age (Ismail et al., 2018).

De Tobel et al. (2017) proposed an automated technique for age estimation based on the mandibular third molar development using panoramic radiographs by employing pattern recognition and classification approaches to the target images. First, image contrast is normalized for all data and ROIs, which indicate the third molar, were cropped using the Photoshop software. Then, a pre-trained model of the AlexNet network was adopted, and the performance was evaluated in a 5-fold cross-validation scenario, using different validation metrics to obtain the accuracy, Rank-N recognition rate, mean absolute difference, and linear kappa coefficient. As a result, the proposed method can stage lower third molar development according to staging by human observers. But more training is needed with data because the pilot study was only done on 20 images.

Recently, the DCNN has also been introduced to perform automated tooth segmentation, in which the segmentation is done automatically. This type of ML has shown better performance compared to other mathematical approaches. Matsuda et al. (2020) improved as proposed in De Tobel et al. (2017). Instead of using the raw images as input data to the CNN architecture, the images were imported to Adobe Photoshop CC 2018 and segmented using the built-in tools before the classification process, using the DenseNet201 network for automatic stage allocation. The authors hypothesized that segmenting only the third molar could improve automated stage allocation performance based on the improvement. Another update on this research was proposed by Banar et al. (2020), which aims to develop a fully automated system to stage the third molar development. By providing the ground truth images, which are manually segmented as described in Merdietio Boedi et al. (2020), three main steps are proposed; third molar localization, segmentation, and classification. Image localization involves the prediction of the geometrical centre within the ground truth image cell using a YOLO-like (Redmon et al., 2016) CNN architecture, and the ImageNet (Russakovsky et al., 2015) pre-trained modal was employed to detect the rectangular ROI, which is the location of the third molar itself presented on the original input image. Then, another CNN was employed to segment the extracted ROI. A final CNN combines the third molar's ROI and segmentation to classify the third molar's developmental stage using two different CNN architectures: a simple CNN with ten layers and the more complex DenseNet201 (Huang et al., 2017), as proposed by Merdietio Boedi et al. (2020). Also, the authors said that future research should include the steps for estimating age instead of focusing on the proposed three-step procedure since this step was not included in the proposed framework.

Another study which utilized the third molar to perform the automatic developmental stage assessment has been proposed by Upalananda et al. (2021). In this study, third molar images in every developmental stage were segmented manually. The pre-trained GoogLeNet (Szegedy et al., 2015) architecture was employed. The stochastic gradient descent (SGD) function was used for training, with the hyperparameters set to default: learning rate of 0.001, training epochs of 10, and mini-batch size of 32. The authors reported that there was inconsistency in accuracy across developmental stages. For example, early developmental stages (Stages D and E) had higher accuracy, whereas later developmental stages had less accuracy (Stages F to H). This is because of the morphological variety of dentition at each stage of development, which grows in complexity as it approaches completion.

Another recent approach that utilizes the DCNN has been proposed by Lee et al. (2020) and Kahaki et al. (2020). However, concerning dental age estimation, the literature states that the variation of the third molar might affect the accuracy of age estimation in different populations (Tafrount et al., 2019). As such, regarding the automated approach, the classification accuracy may also be affected due to the morphology of the tooth and its surroundings. In addition, the unwanted ROI, such as periodontal ligament, bony structures, and mandibular nerve canal, influences the performance of automated stage allocation, as stated in Merdietio Boedi et al. (2020).

Variations in dental morphology may affect the performance of the automated system. For example, previous research reported that monoradicular teeth are more resistant to destruction. Besides, in terms of morphological appearance, they had excellent morphology associated with large pulpal areas present in the digital panoramic dental radiographs compared to incisors (Olze et al., 2012). Meanwhile, the superimposition of the normal dentition will superimpose the vertebrae in the centre of the panoramic image. Thus, they are prone to poor intensity, especially in the middle area of the image, due to the ghost image appearance of the spine and the bite-blocker effect of the x-ray procedure. Besides, molar teeth have a more complicated morphology, consisting of two or three roots, each containing one or two root canals. Moreover, as the root formation reaches approximately one-third of its development, the formation of inter-radicular bifurcation begins where this structure looks like a small clip has appeared in the lower-middle area of the tooth. Typically, these clips are not attached to the main object, which is the tooth structure itself. From a computer vision point of view, the image may have two connected parts: the main structure of the tooth and the clips. This makes it hard for the ML algorithm to pull out the features.

Despite the complicated morphological structure, the first molar is considered to be the most reliable tooth for estimating dental age (Shah and Venkatesh, 2016), Kim et al. (2021) developed a CNN model to determine an individual's age group by extracting the image patches consisting of all four first molar from the panoramic radiographs. First, the CNN architecture of ResNet152 (He et al., 2016) was employed, where the network weights were initialized using pre-trained weights from the ImageNet dataset. Then, according to the age and location of the tooth, the learned features of CNNs were visualized as a heatmap, which demonstrated that CNNs focus on various anatomical parameters, such as tooth pulp, alveolar bone level, or interdental space.

Meanwhile, Mohammad et al. (2021) proposed the deep neural networks associated with the pre-trained model called AlexNet to classify the first and second mandibular premolar teeth. Based on the original training dataset, significant ME was found in stage D of dental development over 1.0 years. Therefore, the authors proposed five new sub-stages to reduce the discrepancy: D1, D2, D3, D4, and D5. Then, the ratio of true classification to total observations is obtained using the same pre-trained model. AlexNet results in 92.5% of classification accuracy. An advanced DL approach has been proposed by the same authors, in which Chollet (2015) and TensorFlow Developers (2022) were used to classify dental developmental stages (Mohammad et al., 2022). In this research, a DL model was built from scratch. The robust model achieved an accuracy of 97.74, 96.63, and 78.13% on the training, validation, and testing sets, respectively. A customized CNN model indeed increased the performance of human identification (Mohammad et al., 2020). An automatic human identification system (DENT-net) (Fan et al., 2020) and a Learnable Connected Attention Network (LCANet) (Lai et al., 2020) are other examples of the recent customized model. These two models employed the same loss function, cosine loss, in which their approach achieves a more competitive result than the other losses function across their dataset.

A recent publication that utilized the largest panoramic dental X-ray image dataset in forensic odontology literature was trained on deep neural networks proposed by Milošević et al. (2022). This study aims to verify the deep neural network in solving age estimation problems. The authors successfully verified the literature for estimating the age of adult and senior subjects by using one of the most extensive datasets stated in the literature. The proposed CNN architecture consists of four parts. The first part is applying a pre-trained CNN for feature extraction where pre-trained models of DenseNet201, VGG16, InceptionResNetV2, ResNet50, VGG16, VGG19, and Xception were tested. The second part of the structure was a 1 × 1 convolutional layer used to adjust the number of channels in the final feature map. In contrast, the third part involved the optional attention mechanism, and the last part consisted of two fully connected layers. Finally, hyperparameter tuning was done to obtain the best performance of a model on a dataset. Based on the hyperparameter optimization, VGG16, with 40 channels in the final convolutional layer and 128 units in the second to last fully connected layers, with no attention mechanism and batch normalization, exhibits a high-performing model. In many articles, scholars rarely mention hyperparameter optimization to generate the optimal CNN model. However, as this step is essential in regulating ML behavior, this new publication can be used as a reference. Before this publication, Merdietio Boedi et al. (2020) reported that VGG16 could achieve the highest accuracy among those six CNN architectures, even with a small dataset, by employing two transfer learning methods: pretraining and fine-tuning. The ImageNet dataset, a large-scale image recognition dataset including over 14 million labeled images, has been utilized in both transfer learning methods.

#### Dental comparison

The establishment of an individual's identity is an essential aspect of forensic identification. In a large-scale disaster, forensic teams are challenged to perform effective and efficient identification by analyzing available human identifiers (Kurniawan et al., 2020). Human dentition is one of the recommended primary identifiers by Interpol. The scientific basis for dental identification is the comparison of antemortem and postmortem data based on the unique characteristics of human dentition. A previous study explained that the number and complexity of dental restorations increased with age (Andersen et al., 1995). In forensic dental identification, an individual who had a number of complicated dental treatments was easier to identify than someone who had little or no treatment (Pretty and Sweet, 2001a). Dental treatment patterns are regarded as a distinct and powerful feature that represents an individual's identity.

The application of AI in forensic dental identification can help to achieve a more efficient and effective identification process (Putra et al., 2022). The DL methods such as CNN and R-CNN have been developed and used for automatic tooth detection on dental radiographs to support individual identification (Miki et al., 2017; Chen et al., 2019; Mahdi et al., 2020). Choi et al. (2022) conducted a study of the automatic detection of teeth and dental treatment patterns on OPG using deep neural networks. The detection of natural teeth and dental treatment was done with a pre-trained object detection network, which was a CNN modified by EfficientDet-D3. The study reported the outstanding performance of CNN in automatic detection of natural teeth (99.1% precision), prostheses (80.6%), treated root canals (81.2%), and implants (96.8%).

A study by Heinrich et al. (2018) proposed an automatic comparison between antemortem and postmortem panoramic radiographs using computer vision. According to the findings of this study, the proposed technique could be a reliable method for comparing antemortem and postmortem OPG with an average accuracy of 85%. The systematic matching yielded a maximum of 259 corresponding points for successful identification between two different OPGs of the same person and a maximum of 12 points for other non-identical people. The challenge of the study by Heinrich et al. is associated with the identification of a person with only a few teeth or no special characteristics, such as dental fillings, dental implants, and prostheses. The inability to identify can be attributed to a lack of quality of OPG, as dental characteristics could not be extracted sufficiently from an overexposed radiograph. This study suggests that computer vision enables automated identification with short computation and reliable results.

### Strength of the study

To our knowledge, this is the most recent comprehensive scoping review of AI technology in forensic odontology. Existing review articles have discussed AI technology published in January 2000 up till June 2020, which focused on the three potential applications of AI-based methods in forensic odontology, such as human bite marks, sex estimation and age estimation, except for the application of AI in dental comparison where this may result in omission of potential research articles that related to this field of study. In addition, this scoping review has screened publicly accessible resources worldwide based on the designated inclusion and exclusion criteria. Although this study was conducted as a scoping review, it followed a structured methodology that includes implementing AI technology in forensic odontology-related study factors, algorithm intervention, performance, and research. Therefore, this article may be the most recent study contributing to AI technology in forensic odontology.

### Knowledge gaps

This article demonstrates shortcomings and a significant knowledge gap in prior research on AI technology in forensic odontology. Our review confirms the research community's interest in human identification assisted by ML architecture. However, the following constraints and challenges must be addressed for future development. First, studies that focused on transfer learning or using pre-trained models overestimate the estimation performance compared to its performance in real-world problems involving intra- and inter-observer agreement. In terms of the application of AI in age estimation, most studies applied staging techniques that considered morphological dental development, enabling human observers to compare the automated system and manual staging. However, comparison with human observers is unable when it comes to continuous data, which involves morphometric parameters such as volumes or the length of the anatomical structures.

In addition, studies involving preprocessing using several computer vision algorithms for segmentation exhibit multiple performance metrics that restrict the comparison of the reported study. For example, model accuracy was not the right metric to evaluate the performance of the segmentation algorithm. The appropriate metric to evaluate the method involving object segmentation was the intersection over union (IoU). Unfortunately, many studies have overlooked this metric that proposed object segmentation before classification. With the improperly reported details on the performance metric, the classification of the segmented ROI may remain doubtful. To address this issue, researchers are advised to report their research with diverse performance metrics that is significant to the proposed methodology.

This review shows a significant increase in publications over the previous 2 years, indicating a significant rise in knowledge, recognition, trends, and interest in using AI technology in forensic odontology for human identification. However, much of the existing literature is focused only on age and sex estimation, which uses dental radiographs to identify individuals. Hence, the potential application of AI technology in this field of study was restricted. Additionally, this study demonstrates that most of the architecture algorithm employed was based on the transfer learning approach, which uses the pre-trained model as the starting point for a model on a new task. As we know, transfer learning is an option for the small sample of the dataset.

Meanwhile, there is not much study developing a new ML model from scratch, which somehow may be an alternative to research which has limited Additionally, this study demonstrates that most of the architecture algorithm employed was based on the transfer learning approach, which uses the pre-trained model as the starting point for a model on a new task. As we know, transfer learning is an option for the small sample of the dataset. Meanwhile, few studies are developing a new ML model from scratch, which may be an alternative to research with a limited dataset.

## Conclusion and future recommendations

Machine learning has proved its capability to predict as humans do. Therefore, ML's feasibility in the field of forensic odontology is undeniable. Furthermore, the reviewed articles show that ML techniques are reliable for studies that involve continuous features, such as morphometric parameters, that require fewer training datasets to be trained on the ML model with promising outcomes. Meanwhile, DL networks learn by observing complex patterns in the data they experience. Hence, large datasets may be required to be trained on the network as they need to learn as many features as possible to make a good prediction. In the meantime, this approach has been a significant success in the ML field due to its ability to learn task-specific feature representations automatically. Hence, it is one of the most frequently utilized types of ML in many applications nowadays.

Based on the presented studies, it is evident that AI technology has been successfully implemented in various aspects of forensic odontology. However, it is essential to highlight that all the studies published to date and reviewed in this present study were based on secondary data, which could not provide the actual performance of AI in real-world problems. Hence, in the future, the real dataset acquired from the criminal cases found at the scene or from the actual real-life incident, such as a mass disaster, was recommended to be tested on the proposed ML architecture, and a comparison should be made between these two approaches. In addition, instead of reporting the performance of the proposed method using simple per cent agreement calculation, Cohen's kappa coefficient is recommended as it is more robust that applies the statistic to measure inter-rater reliability. This can be done by introducing a cross-tabulation based on the result of the testing dataset, which includes two different raters: the computer prediction result and the other from a human observer.

## Data availability statement

The original contributions presented in the study are included in the article/supplementary material, further inquiries can be directed to the corresponding author.

## Author contributions

NM designed the study and drafted the manuscript, which was revised and completed by RA, AK, and MM. All authors have approved the final version of the manuscript for submission.

## Funding

This work was supported by the GPK Research Grant, Universiti Teknologi MARA [600-RMC/GPK 5/3 (188/2020)].

## Conflict of interest

The authors declare that the research was conducted in the absence of any commercial or financial relationships that could be construed as a potential conflict of interest.

## Publisher's note

All claims expressed in this article are solely those of the authors and do not necessarily represent those of their affiliated organizations, or those of the publisher, the editors and the reviewers. Any product that may be evaluated in this article, or claim that may be made by its manufacturer, is not guaranteed or endorsed by the publisher.
